# Training load and fitness monitoring in Czech football: coach practices and perspectives

**DOI:** 10.3389/fspor.2025.1513573

**Published:** 2025-01-31

**Authors:** Dominik Bokůvka, Michal Hrubý, Kristýna Čuperková, Tomáš Vencúrik, Vitor Padinha, Ana Carolina Paludo

**Affiliations:** ^1^ Department of Sport Performance and Exercise Testing, Faculty of Sports Studies, Masaryk University, Brno, Czechia; ^2^ School of Sport, Polytechnic University, Santarém, Portugal; ^3^ Sport Physical Activity and Health Research Innovation and Technology Center (SPRINT), Santarém Polytechnic University, Santarém, Portugal; ^4^ Comprehensive Health Research Centre (CHRC), School of Health and Human Development, University of Évora, Évora, Portugal

**Keywords:** football, training, team sports, performance, workload

## Abstract

**Introduction:**

The study aimed to describe the practices and perspectives of Czech football coaches regarding the monitoring of players’ training load and physical performance, with a focus on identifying key barriers and preferred sources of information.

**Methods:**

A total of 235 football coaches completed an online survey comprehending training load monitoring methods, physical performance assessments, barriers to implementation, and information sources.

**Results:**

Among respondents, 93.7% reported monitoring training load, with training diaries (70%) being the most utilized method for external load measures and heart rate (45%) for internal load. Despite this, 42.7% of coaches did not monitor internal load and 21.7% did not conduct physical fitness evaluations. The most frequently reported barrier was a lack of resources (74.5%), though elite-level coaches (52.8%) and strength and conditioning coaches (75%) identified human resources as their primary limitation. Across all levels, the Football Association was the preferred source of information (61.7%).

**Conclusion:**

The findings highlight the predominance of traditional monitoring practices among the Czech football coaches, alongside with notable gaps in internal load tracking and fitness evaluation. The resource constraints remain a major barrier. Practical recommendations include promoting economical monitoring tools, such as RPE, and enhancing collaboration among stakeholders to improved monitoring strategies. The Football Association's play a key role on support these efforts.

## 1 Introduction

In the field of sports science, there is a growing emphasis on monitoring training load and physical fitness to understand fatigue, optimize performance and prevent injuries in athletes (1–4). Nonetheless, despite the well-established importance of training load monitoring, there remains a gap between academic knowledge and its implementation in practice, particularly among football coaches (5–8). Some topics and methodologies recommended by scientists often lack practicality or direct application in the field, conflicting the effective dissemination of academic knowledge to coaches and practitioners. A prior investigation with coaches revealed key areas in sports science they want to learn more about, with load, fitness, and fatigue monitoring emerging as prominent topics (9).

Training load (TL) can be understood as the cumulative stress imposed on an athlete's body during individual or multiple training sessions (10). TL is typically classified into internal and external measures. Internal training load (ITL) can comprehend relative biological stressors imposed on athletes during the training, while external training load (ETL) quantifies the objective workload performed by athletes during the training (1, 3, 11). ITL monitoring involves physiological and psychological parameters, including perceived effort, cardiac autonomic indicators (such as heart rate and heart rate variability), and biochemical markers like lactate concentrations, hormonal levels and immunological responses (1–3, 11). Conversely, ETL evaluation is related to the organization of the training, including type, intensity, and volume of exercises, incorporating aspects like power, speed, acceleration, time-motion, and neuromuscular function (1–3, 11). Concurrently with load monitoring, evaluating athletes’ physical fitness stands as an important parameter to track training and match efficacy, ensuring peak performance and minimizing the risk of injury.

Despite the assortment of markers available for monitoring training load in athletes, coaches often face barriers that limit their ability to implement it effectively. It has been highlighted elsewhere perceived barriers to training load monitoring effectiveness in elite high-performance football, with limited human resources scoring highest, followed by coach buy-in (12). Recognizing these potential challenges has underscored the significance of sports scientists disseminating information about the training process through more accessible channels for coaches and practitioners, such as diverse media formats (e.g., free downloads, simplified versions published in coaching journals, book chapters, and infographics) (6). Sometimes, comprehension barriers and time constraints can impede coaches and practitioners from accessing information from academic sources (13).

Understanding the practices and perspectives of training load monitoring among coaches is crucial for point out the challenges faced on the pitch. This insight not only improves the existing literature on the topic but also contributes to potential solutions in both academic and organizational settings. Building on previous research investigating training load monitoring practices and perspectives in professional clubs competing in top-tier leagues (12, 14), this study specifically examines the approaches and viewpoints of football coaches in the Czech Republic. The study followed three specific aims: (i) describe the methods used by coaches to monitor training load and physical fitness; (ii) investigate potential barriers to effectively monitoring training load; (iii) verify coaches' preferred sources of information on training load methods. It is hypothesized that Czech football coaches primarily rely on traditional methods for training load monitoring, and resource constraints are the primary barriers limiting advanced TL practices.

## 2 Materials and methods

### 2.1 Participants

An online survey was distributed to coaches registered at the Football Association of the Czech Republic (FAČR). A total of 235 participants completed the entire survey. Inclusion criteria were to be part of FAČR and coach a football team in the Czech Republic of categories higher than U-16. The sample size for the present study follows the previous one with a similar design and purpose in coaches from football (14). All participants signed informed consent stating their voluntary participation. The study was approved by the University of Masaryk University Ethics Committee (EKV-2022-054).

The average age of participants was 43.8 years. The sample consisted predominantly of male coaches (97%), with only six female participants (3%). Despite the low representation of female coaches, their inclusion on the study provides information into underrepresented perspectives in Czech football coaching.

### 2.2 Study design

The study presents a cross-sectional design to investigate Czech football coaches' monitoring practices and perspectives regarding training load and physical fitness evaluation. A survey was developed to cover the study's aims and administered online. Before the data collection, with the consent of the FAČR, the research team promoted an online meeting with coaches associated with FAČR to explain the main aim of the study and answer possible questions. Subsequently, the survey link (built on Google Forms) was disseminated to coaches via email linked with the FAČR. The survey was opened for answers during a 2-week period (March 2024).

### 2.3 Procedure

The survey was specifically developed for the coaches in the Czech language, based on extant literature (8, 14, 15) and the research team's expertise on the topic of football coaching. It was structured to address three primary objectives: (i) describe the methods coaches use to monitor training load and physical fitness; (ii) investigate potential barriers to effectively monitor training load; (iii) verify coaches’ preferred sources of information on training load methods. After finishing the first draft, the authors independently revised and adjusted the survey when necessary. Comprising 23 items, the survey predominantly featured closed-ended questions (e.g., Likert scale), to facilitate the participants' answers. Most questions incorporate the option “other”, allowing respondents to provide additional responses not listed. Five-section were created as follows:

#### 2.3.1 Section 1: coaches’ demographic characteristics

Demographic information about the coaches' age, education, experience, level and work categories were asked by mostly closed-ended questions. Coaches were separated into positions based on their role in the team: head coach, assistant coach, strength and conditioning coach, and others (job roles different from those proposed). Additionally, to determine the coaches' classification, the study considered the competitive level of the teams they were coaching, as indicated elsewhere (14). Accordingly, coaches were categorized into elite (male: senior top tier and second tier, U19 or U17 top tier; female: senior top tier), high performance (male: senior third tier, U19 or U17 second tier; female: senior second tier, U19 or U17 first tier) and amateur (male: senior fourth tier and lower, U19 and U17 third tier and lower; female: senior third tier and lower, U19 and U17 second tier and lower) levels.

#### 2.3.2 Section 2: monitoring training load and physical fitness

Consisting of nine closed-ended and multiple-choice questions, this survey section had questions regarding physical fitness evaluation as well as the frequency, variables, methods and data storage used by the respondents to monitor external and internal training load. All questions had an option “other”, allowing the respondent to write an additional response that was not mentioned.

#### 2.3.3 Section 3: barriers to monitoring training load

The survey included a multiple-choice question about the primary barriers perceived by the coaches regarding monitoring training load. This question aimed to elucidate the challenges perceived by respondents in load monitoring. It presented four predefined options, supplemented by an “other” choice, allowing participants to specify additional barriers not addressed within the provided options.

#### 2.3.4 Section 4: primary sources of information on training load monitoring

It included a multiple-choice question exploring the primary preferred sources for searching for information utilized by the coaches for monitoring training load. This question offered four predefined options, along with an “other” choice, enabling the respondents to specify additional sources of information not listed among the provided options.

#### 2.3.5 Section 5: additional information

At the end of the survey, an optional question was included for respondents: *Is there anything related to load monitoring in football that we haven't asked about, but that you would like to mention?*

The complete survey version can be accessed in the Supplementary Material section (S1).

### 2.4 Statistical analysis

Considering the nature of the study, descriptive frequency analysis was conducted to profile the participants. Findings for each survey question were presented as absolute frequency counts and percentage of respondents. Where relevant, results were aggregated to represent the percentage of respondents within specific group (e.g., coach level and role), enhancing the interpretability of the data. All analyses were performed using JAMOVI (version 2.3.28) software and graph visualizations on GraphPad (version 5.0).

## 3 Results

Overall, 235 respondents completed the survey. The majority of the respondents were head coaches (78.3%), male (97%), with an average age of 43.8 years old, working with only one team category (84%). Additionally, among the respondents, most of them worked in the adult male category (25.8%), with experience in the category of up to 4 years (45.5%) and do not have a degree in sports science or related discipline (82.6%). Considering the female respondents (*n* = 6; 38.4 years), the majority had a UEFA B license (50%), worked as a head coach (66.7%), on amateur teams (50%) and coached only male teams (50%). More detailed sociodemographic data are reported in (Supplementary Material S2, Table 1).

**Table 1:** The proportion of respondents related to age categories, competition level worked with, and license (*n* = 235).

	Head coach (*n* = 184)	Assistant coach (*n* = 38)	SC coach (*n* = 3)	Other (*n* = 10)
	M/F (%)	M/F (%)	M/F (%)	M/F (%)
Age categories worked with				
U16	48/11 (23.7%)	8/1 (15.2%)	0/0 (0%)	5/1 (20.7%)
U17	37/6 (17.3%)	11/0 (18.6%)	1/0 (16.7%)	6/1 (24.1%)
U18	22/6 (11.2%)	7/0 (11.9%)	0/0 (0%)	4/0 (13.8%)
U19	46/3 (19.7%)	11/2 (22.1%)	2/0 (33.3%)	6/1 (24.1%)
Adult	65/5 (28.1%)	18/1 (32.2%)	2/1 (50%)	4/1 (17.2%)
Competition level (league) worked with				
Elite	8 (3.8%)	7 (20.5%)	3 (100%)	6 (60%)
High performance	27 (14.8%)	7 (17.9%)	0	1 (10%)
Amateur	148 (80.9%)	24 (61.5%)	0	3 (30%)
Not specified	1 (0.5%)	0 (0%)	0	0
Coach license				
UEFA Pro	18 (9.8%)	0 (0%)	0 (0%)	0 (0%)
UEFA A	65 (35.3%)	16 (42.1%)	0 (0%)	3 (30%)
UEFA B	96 (52.2%)	22 (57.9%)	1 (33.3%)	6 (60%)
UEFA C	2 (1.1%)	0 (0%)	0 (0%)	0 (0%)
FAČR C	3 (1.6%)	0 (0%)	0 (0%)	0 (0%)
Other^a^	0 (0%)	0 (0%)	2 (66.7%)	1 (10%)

SC, strength and conditioning; UEFA, Union of European Football Association; FAČR, Football Association of the Czech Republic.

^a^ Other license (goalkeeper license A, UEFA fitness, fitness trainer FAČR).

Table 1 categorized respondents into four distinct roles: head coach, assistant coach, strength and conditioning coach (SC) and others (e.g., goalkeeper coach (*n* = 6), analytical coach (*n* = 1), two position (*n* = 1), academy head coach (*n* = 2). The majority of head coaches (80.9%) and assistant coaches (61.5%) were involved with amateur teams, whereas all SC coaches worked exclusively with elite teams. Notably, UEFA B licenses were held by 52.2% of head coaches 52.2%, 57.9% of assistant coaches, and 60% of respondents in other roles. The SC coaches possessed UEFA B license, goalkeeper license A and UEFA fitness certifications.

### 3.1 Monitoring training load and physical fitness

**Figure 1:**
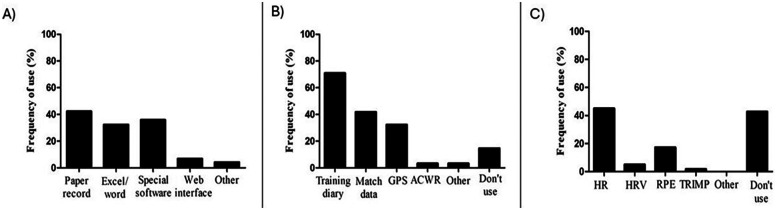
Proportion of respondents about data storage **(A)**, external load monitoring **(B)** and internal load monitoring **(C)**. GPS, global position system; ACWR, acute: chronic workload ratio; HR, heart rate; HRV, heart rate variability; RPE, rate of perceived exertion; TRIMP, training impulse.

Overall, the survey results indicate that the majority of respondents reported monitoring training load using objective (*n* = 142; 60.4%) and subjective measures (*n* = 78; 33.2%), in each training and match (*n* = 124; 56.5%). Figure 1 illustrates the frequency with which respondents utilized the parameters for monitoring training load. Regarding data storage practices, among those respondents who reported monitoring training load (*n* = 220), there was more frequent use of paper records and special software (Figure 1A).

When monitoring external load, training diaries emerged as the most commonly used tool (Figure 1B). At the same time, heart rate (HR) monitoring predominates for assessing internal load, with 45% of respondents utilizing this method and 42.7% opting not to monitor internal load (Figure 1C). Biochemical parameters were used by only 3.8% of respondents, with 1.7% incorporating lactate measurements. Regarding the physical fitness evaluation, endurance and speed tests were the most frequently employed at 57.4% and 48%, respectively. Notably, 21.7% of respondents indicated that they did not use any formal tests for physical fitness evaluation (see Supplementary Material S2, Table 2).

**Table 2:** Load monitoring and physical fitness evaluation from coaches, separated by respondent position in the football teams.

	Head coach (*n* = 184)	Assistant coach (*n* = 38)	SC Coach (*n* = 3)	Other (*n* = 10)
Yes	110 (59.8%)	24 (63.1%)	3 (100%)	5 (50%)
No	12 (6.5%)	1 (2.6%)	0	2 (20%)
Yes, subjectively^a^	62 (33.7%)	13 (34.2%)	0	3 (30%)
Frequency of load monitoring				
Each training and match	94 (54.6%)	23 (62.2%)	3 (100%)	4 (50%)
Only on matches	30 (17.4%)	6 (16.2%)		1 (12.5%)
Only on training	39 (22.7%)	7 (18.9%)		1 (12.5%)
Other^b^	9 (5.2%)	1 (2.7%)		2 (25%)
Data storage				
Paper record	74 (35.9%)	18 (39.1%)	0 (0%)	1 (10%)
Excel/Word	57 (27.7%)	9 (19.6%)	2 (40%)	3 (30%)
Special Software	54 (26.2%)	17 (36.9%)	2 (40%)	6 (60%)
Web interface	13 (6.3%)	1 (2.3%)	1 (20%)	0 (0%)
Other^c^	8 (3.9%)	1 (2.3%)	0 (0%)	0 (0%)
Monitoring external load				
Training dairy	129 (45.6%)	24 (38.7%)	1 (16.7%)	2 (14.3%)
Match data	71 (25.1%)	16 (25.8%)	1 (16.7%)	4 (28.6%)
GPS	46 (16.2%)	17 (27.9%)	3 (50%)	5 (35.7%)
ACWR	3 (1.1%)	1 (1.6%)	1 (16.7%)	2 (14.3%)
Don't use	27 (9.5%)	4 (6.4%)	0 (0%)	1 (7.1%)
Other^d^	7 (2.5%)	0 (0%)	0 (0%)	0 (0%)
Monitoring internal load				
HR	80 (42.1%)	16 (36.4%)	0 (0%)	3 (33.3%)
HRV	8 (4.2%)	2 (4.5%)	0 (0%)	1 (11.1%)
RPE	27 (12.2%)	8 (18.2%)	1 (33.3%)	2 (22.2%)
TRIMP	4 (2.1%)	0 (0%)	0 (0%)	0 (0%)
Don't use	71 (37.4%)	18 (40.9%)	2 (66.7%)	3 (33.3%)
Biochemical analysis				
Lactate	4 (2.3%)	0 (0%)	0 (0%)	0 (0%)
Glucose	1 (0.6%)	0 (0%)	0 (0%)	0 (0%)
Hemoglobin	1 (0.6%)	0 (0%)	0 (0%)	0 (0%)
Hormone	2 (1.1%)	1 (2.6%)	0 (0%)	0 (0%)
Don't use	168 (95.4%)	37 (97.4%)	3 (100%)	8 (100%)
Physical test				
Speed	89 (22.2%)	19 (22.3%)	1 (11.1%)	4 (14.3%)
Strength	66 (16.4%)	12 (14.1%)	3 (33.3%)	7 (25%)
Endurance	108 (26.9%)	21 (24.7%)	2 (22.2%)	4 (14.3%)
Flexibility	30 (7.5%)	10 (11.8%)	1 (11.1%)	5 (17.8%)
Coordination	32 (8%)	6 (7.1%)	0 (0%)	2 (7.1%)
Testing Battery	14 (3.5%)	3 (3.5%)	0 (0%)	3 (10.7%)
Motor testing FAČR	19 (4.7%)	5 (5.9%)	2 (22.2%)	2 (7.1%)
Don't use	41 (10.2%)	9 (10.6%)	0 (0%)	1 (3.6%)
Other	2 (0.5%)	0 (0%)	0 (0%)	0 (0%)

SC, strength and conditioning; FAČR, Football Association of the Czech Republic; GPS, global position system; ACWR, acute: chronic workload ratio; HR, heart rate; HRV, heart rate variability; RPE, rate of perceived exertion; TRIMP, training impulse; FAČR, football federation from Czech Republic.

^a^ Monitoring “by eye”.

^b^ Other types of monitoring frequency (irregularly, subjectively, once in 2–3 months, once a year, during pre-season, after an intense session or after a player returns from injury).

^c^ Other types of data storage (in my head, by guessing, communication with the payers).

^d^ Other external load monitoring (video from the match to see the intensity, Balke's test, smartwatch).

Table 2 displays the results of training load monitoring and physical fitness evaluation according to the coach's role in the team. On average, all SC coaches don't monitor players' internal load, only a small percentage of the head coaches (4.6%) use biochemical parameters. Interestingly, 10% of head coaches and assistant coaches don't use any test to assess the players' physical fitness.

Table 3 summarizes the results based on the respondent's coaching levels. Amateur team coaches reported a high reliance on subjective load monitoring (40%), paper records for data storage (43.5%), training diaries for the external load monitoring (47.4%) and notable lack of physical fitness testing (13.4%) compared to elite and high-performance counterparts. In contrast, elite coaches demonstrated greater use of advanced tools, with 63.3% utilizing specialized software for data storage and 38.8% employing GPS for monitor external load monitoring, though 48% reported not monitoring internal load—a higher percentage than both amateur and high-performance coaches.

**Table 3:** Load monitoring and physical performance evaluation from coaches, separated by level of coaching team competition.

	Elite (*n* = 24)	Sub-elite (*n* = 35)	Amateur (*n* = 175)
Yes	21 (87.5%)	29 (82.9%)	91 (52%)
No	0	1 (2.8%)	14 (8%)
Yes, subjectively^a^	3 (12.5%)	5 (14.3%)	70 (40%)
Frequency of load monitoring			
Each training and match	20 (83.3%)	28 (82.3%)	75 (46.6%)
Only on matches	2 (8.3%)	3 (8.8%)	32 (19.9%)
Only on training	2 (8.3%)	1 (2.9%)	44 (27.3%)
Other^b^	0 (%)	2 (5.9%)	10 (6.2%)
Data storage			
Paper record	1 (3.33%)	7 (16.7%)	84 (43.5%)
Excel/Word	7 (23.3%)	13 (30.5%)	51 (26.4%)
Special Software	19 (63.3%)	16 (38.1%)	43 (22.3%)
Web interface	3 (10%)	5 (11.9%)	7 (3.6%)
Other^c^	0 (%)	1 (2:4%)	8 (4.1%)
External load			
Training dairy	12 (24.5%)	31 (40.3%)	112 (47.4%)
Match data	13 (26.3%)	23 (29.9%)	56 (23.7%)
GPS	19 (38.8%)	21 (27.3%)	30 (12.7%)
ACWR	3 (6.1%)	2 (2.6%)	1 (0.4%)
Don't use	2 (4.1%)	0 (0%)	30 (12.7%)
Other^d^	0 (0%)	0 (0%)	7 (3%)
Internal load			
HR	10 (40%)	18 (42.8%)	71 (40.1%)
HRV	1 (4%)	2 (4.8%)	8 (4.5%)
RPE	2 (8%)	8 (19%)	27 (15.2%)
TRIMP	0 (0%)	0 (0%)	3 (1.7%)
Don't use	12 (48%)	14 (33.3%)	68 (38.4%)
Biochemical analysis			
Lactate	2 (8%)	1 (2.9%)	0 (%)
Glucose	0 (%)	0 (%)	0 (%)
Hemoglobin	0 (%)	0 (%)	0 (%)
Hormone	1 (4%)	0 (%)	2 (1.2%)
Don't use	22 (88%)	33 (97%)	161 (98.8%)
Physical test			
Speed	14 (16.7%)	24 (22%)	75 (22.8%)
Strength	19 (22.6%)	20 (18.3%)	49 (14.9%)
Endurance	14 (16.7%)	27 (24.8%)	94 (28.6%)
Flexibility	12 (14.3%)	13 (11.9%)	21 (6.4%)
Coordination	6 (7.1%)	8 (7.3%)	26 (7.9%)
Testing Battery	6 (7.1%)	3 (2.7%)	10 (3%)
Motor testing FAČR	10 (11.9%)	10 (9.2%)	8 (2.4%)
Don't use	3 (3.6%)	4 (3.7%)	44 (13.4%)
Other	0 (0%)	0 (0%)	2 (0.6%)

SC, strength and conditioning; FAČR, Football Association of the Czech Republic; GPS, global position system; ACWR, acute: chronic workload ratio; HR, heart rate; HRV, heart rate variability; RPE, rate of perceived exertion; TRIMP, training impulse; FAČR, football federation from Czech Republic.

^a^ Monitoring “by eye”.

^b^ Other types of monitoring frequency (irregularly, subjectively, once in 2–3 months, once a year, during pre-season, after an intense session or after a player returns from injury).

^c^ Other types of data storage (in my head, by guessing, communication with the payers).

^d^ Other external load monitoring (video from the match to see the intensity, Balke's test, smartwatch).

**Figure 2:**
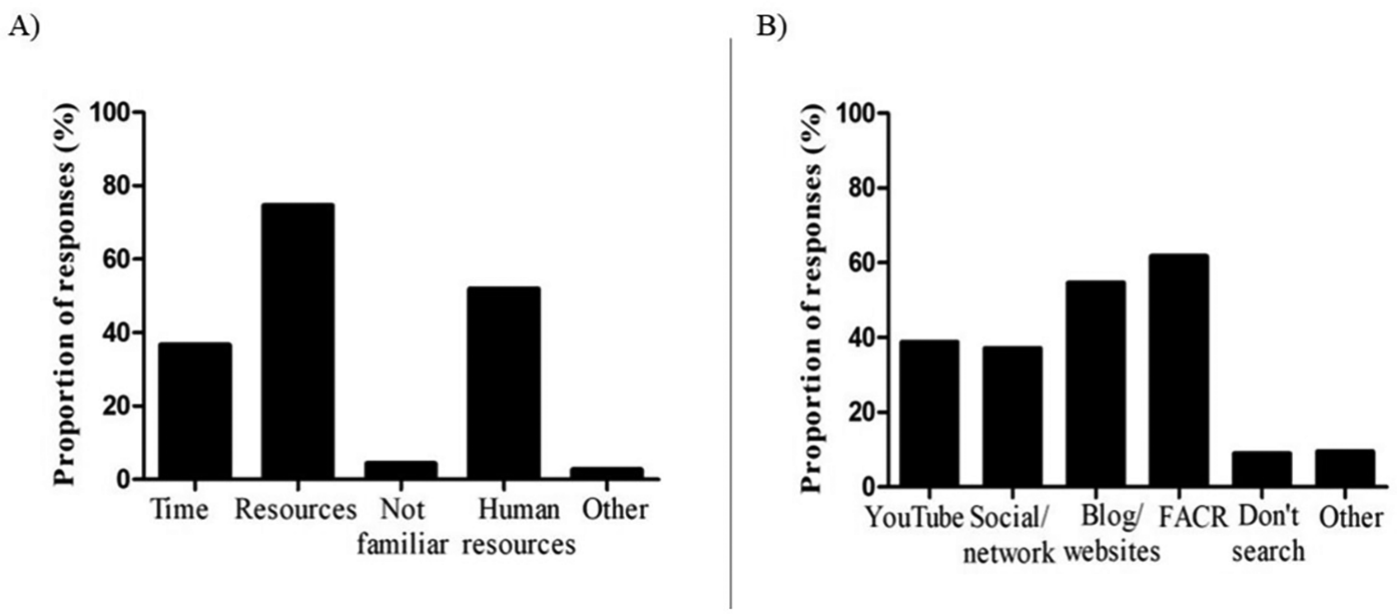
Proportion of respondents about barriers to monitoring training load **(A)** and primary source of information seeking by the respondents about training load **(B)**.

Figure 2 provides the respondents' responses regarding the primary barriers to monitoring training load as well as the primary source of information on monitoring training load they searched for.

### 3.2 Barriers to monitoring training load

Resource constraints were the most reported barrier (74.5%), with variations across the coaching levels and roles. Elite coaches primarily cited human resource limitations (52.8%), while amateur coaches emphasized equipment and financial challenges. Among coaching roles, head coaches (46.4%) and assistant coaches (41.2%) identified resource availability as their main obstacle, whereas SC coaches (75%) and others highlighted human resource issues (Table 4, Figure 2A).

**Table 4:** Barriers to monitoring training load, separated by respondents’ position in the football teams and level of coaching team competition.

	Head coach (*n* = 184)	Assistant coach (*n* = 38)	SC coach (*n* = 3)	Other (*n* = 10)
Time	63 (20.3%)	17 (23.5%)	1 (20%)	5 (33.3%)
Resources^a^	144 (46.4%)	28 (41.2%)	1 (20%)	2 (13.3%)
Not familiar	7 (2.2%)	2 (2.9%)	0 (0%)	1 (6.7%)
Human resources	91 (29.3%)	22 (32.3%)	3 (75%)	6 (40%)
Other^b^	5 (1.6%)	0 (0%)	0 (0%)	1 (6.7%)
	Elite (*n* = 24)	High performance (*n* = 35)	Amateur (*n* = 175)	
Time	5 (13.9%)	10 (17.2%)	71 (23.4%)	
Resources^a^	10 (27.8%)	29 (50%)	135 (44.5%)	
Not familiar	1 (2.8%)	0 (0%)	9 (3%)	
Human resources	19 (52.8%)	19 (32.7%)	83 (27.4%)	
Other^b^	1 (2.8%)	0 (0%)	5 (1.6%)	

SC, strength and conditioning.

^a^ Resources = equipment.

^b^ Other barriers (financing is poor; lack of knowledge about concept and guidelines; not barrier).

### 3.3 Primary sources of information on training load monitoring

The FAČR emerged as the primary channel for accessing information on training load monitoring, reported by 61.7% of respondents. Blogs and websites followed closely, utilized by 54.5%, with YouTube (38.7%) and social networks (37%) also serving as significant sources (Figure 2B). Notably, this information-seeking pattern was consistent across coaching levels and role, reflecting a similar resources. In the “other” category, coaches mentioned academic books, peer-reviewed articles, and internal educational initiatives, such as internships at other clubs or abroad, as alternative sources (Table 5).

**Table 5:** Source from information about monitoring training load, separated by respondents’ position in the football teams and level of coaching team competition.

	Head coach (*n* = 184)	Assistant coach (*n* = 38)	SC coach (*n* = 3)	Other (*n* = 10)
YouTube	75 (19.3%)	13 (16.9%)	1 (14.3%)	1 (5.5%)
Social networks	67 (17.2%)	17 (22.1%)	1 (14.3%)	2 (11.1%)
Blog, Websites	101 (26%)	21 (27.3%)	2 (28.6%)	4 (22.2%)
FAČR	115 (29.6%)	22 (28.6%)	3 (42.9%)	5 (27.8%)
Not search for	18 (4.6%)	3 (3.9%)	0 (0%)	1 (5.5%)
Other^a^	13 (3.3%)	1 (1.3%)	0 (0%)	5 (27.8%)
	Elite (*n* = 24)	High performance (*n* = 35)	Amateur (*n* = 175)	
YouTube	7 (12.7%)	17 (20.5%)	67 (18.9%)	
Social networks	10 (18.2%)	13 (15.7%)	64 (18%)	
Blog, Websites	15 (27.3%)	24 (28.9%)	88 (24.8%)	
FAČR	19 (34.5%)	25 (30.1%)	101 (28.4%)	
Not search for	1 (1.8%)	0 (0%)	20 (5.6%)	
Other^a^	3 (5.4%)	4 (4.8%)	15 (4.2%)	

SC, strength and conditioning; FAČR, Football Association of the Czech Republic.

^a^ Other sources of information [my own education; experienced colleagues; university books and articles (4x); club internal education; internships in other clubs or abroad (4x); communication with strength and conditioning specialists (4x); in the Czech Republic we are not modern, nobody wants to make the change, so I need to get inspiration abroad; Veo (=video recording)].

## 4 Discussion

The study aimed to explore the training load monitoring practices and perspectives of Czech football coaches. The main findings reveal that while most respondents monitor training load, traditional methods such as paper records and training diaries remain widely used. Elite-level coaches, however, are more likely to utilize specialized software to store data and GPS tracking as a measurement of ETL. Despite this, ITL methods remain underutilized by the coaches.

The most prevalent barrier to effective monitoring of training load was identified as the lack of resources, with elite-level coaches and strength and conditioning coaches particularly emphasizing constraints to human resource. Furthermore, a major portion of respondents indicated the FAČR as their primary channel for seeking information on training load monitoring, highlighting its important role in knowledge dissemination within the football coaching community.

### 4.1 Training load monitoring and physical fitness

The participants in the study were predominantly male head coaches holding UEFA B licenses who primarily coached adult and amateur football teams. Generally, these coaches exhibited a frequent engagement in monitoring training load, even though primarily through simple methods such as training diaries for tracking ETL and relying on paper records for data storage. Despite recent advancements in football technologies, particularly in the domain of monitoring external load parameters and the propagation of specialized software, which aids in training monitoring and leverages machine learning for injury risk prediction (16), adoption among coaches, especially those in amateur teams, remains limited. However, coaches at the elite level and strength and conditioning coaches in our study displayed a greater propensity toward embracing these technological approaches.

Coaching education initiatives could play a significant role in addressing these knowledge gaps and barriers. For instance, offering workshops or certifications focused on affordable and easy-to-implement monitoring techniques, such as RPE, could help no approximate the gap between amateur and elite coaching practices. RPE, in particular, provides a low-cost yet validated method for monitoring training intensity, and could help coaches who currently lack access to advanced technologies.

Differences in the monitoring of ETL parameters were evident among respondents when stratified by coaching level. Elite-level coaches demonstrated a higher propensity for utilizing GPS tracking devices, whereas amateur coaches leaned towards the more traditional method of training diaries. Indeed, using GPS devices has become a prevalent measure of training load among elite football teams (3, 12). The adoption of GPS technology in football has seen exponential growth, offering a robust means to quantify athletes' workload during both training sessions and matches.

This is attributed to its ability to capture locomotion metrics, including accelerations, decelerations, and power (17, 18), with longitudinal monitoring proving instrumental in injury prevention (19). However, the low adoption of advanced monitoring tools like GPS among amateur coaches highlights the financial and educational challenges they face. These limitations suggest a need for specific strategies to increase accessibility and understanding, such as subsidized equipment programs or partnerships with educational institutions to provide affordable training.

Regarding ITL monitoring, the most prevalent method among respondents was heart rate measurement, although a considerable percentage reported not using any method. As documented in the literature, popular ITL monitoring methods in football include heart rate and perceived exertion (RPE) (3, 12, 14). RPE has gained widespread acceptance due to its affordability and validity compared to physiological parameters (20). However, its subjective nature poses a limitation, relying on the athlete's perception. Nevertheless, RPE is a valuable tool to indicate the alignment between coaches' programmed training sessions and athletes' perceived exertion levels. Studies have indicated a higher agreement between coaches and athletes in sessions with moderate and high effort than those with low effort (21).

Moreover, the Weston (2018) (14) study highlighted that even in elite teams, subjective coach perception was the most prevalent method for training load monitoring among coaches, sports scientists, and fitness coaches. Incorporating RPE into coaching education initiatives and emphasizing its practicality could address knowledge gaps among amateur coaches, offering a viable and accessible method for training load monitoring. This approach could help amateur coaches overcome barriers related to cost and lack of technical expertise.

Load monitoring has been extensively explored over time, with recent attention focusing on the need for valid metrics to accurately quantify the intensity of training sessions or matches (22). However, the absence of standardized methods or validated classifications leaves the load monitoring process under the control of individual coaches' understanding. As previously noted, the standardization and classification of measures could significantly improve coaches' confidence in the validity and utility of these methods (3). Nevertheless, selecting an appropriate methodology may be contingent upon logistical considerations within the team's operational framework. The prerogative to determine the suitable monitoring tools or techniques should lie within sports professionals engaged in the field (1).

The study also explored the physical fitness practices employed by coaches, focusing on the types of physical tests utilized. Endurance, strength and speed tests emerged as the most commonly used by the coaches. Physical tests are crucial in football, facilitating individualized training prescriptions, performance monitoring, and injury risk prediction (23). Intriguingly, some coaches indicated not performing physical tests. In a previous study, coaches acknowledged the importance of monitoring athletes' fitness levels; however, they also highlighted the complexity associated with understanding and implementing such testing within practical contexts (9).

Adopting personalized testing batteries could provide valuable guidance to coaches, particularly strength and conditioning coaches, in efficiently applying specific tests to assess different components of fitness. Football-specific tests can be conducted with minimal equipment, administered on a football field, and integrated seamlessly into team warm-ups within a single day (24), improving the possibility of being used by coaches from different levels.

### 4.2 Barriers

The survey findings reported significant challenges coaches faced in monitoring training load, with resource constraints, including equipment availability, identified by 74.5% of respondents. Additionally, 51.9% highlighted human resource limitations, emphasizing the need for competent and sufficient team members to support monitoring efforts. Interestingly, distinctions in the barriers came out based on coaches' positions and competitive levels. Head and assistant coaches primarily cited resource scarcity, while SC coaches and other coaches’ roles emphasized human resource constraints. Moreover, elite coaches focused on human resource challenges, whereas high-performance and amateur coaches highlighted resource limitations, underscoring the diverse contexts within coaching structures and competitive tiers. Similarly, elite practitioners from Akenhead and Nassis's (12) study also reported the limited human resources as the greatest barrier to training load effectiveness.

### 4.3 Sources of information

Lastly, the study also investigates coaches' preferred sources for monitoring training load. The predominance of established governing bodies, such as the Football Association (FAČR), as primary sources highlighted the trust coaches place in the institution. Nevertheless, the findings also pointed out the emerging impact of digital platforms. The embrace of blogs, websites, YouTube, and social networks demonstrated the dynamic and digitally driven sources of knowledge acquisition among coaches. When offering valid and reliable content, social media platforms serve as important tools for facilitating information exchange and learning within the coaching community. Furthermore, including the “other” category in the question provided answers like academic literature and participation in internships, which can support the relevance of both digital and traditional channels for coaches' knowledge enhancement. Sports scientists must disseminate academic concepts regarding training load through social media platforms to promote comprehension among coaches across all levels (6).

### 4.4 Limitation

The study is pioneering in describing the methodologies and viewpoints of football coaches in the Czech Republic regarding training load monitoring. However, some limitations need to be highlighted. The sample was gathered through email dissemination targeting coaches from the Czech Football Association, utilizing a convenience sampling method that may not fully represent all coaching levels and positions. It was possible to notice that there was an overrepresentation of respondents from amateur levels, with a scarcity of SC coaches and female coaches. Additionally, the build the survey with Google Forms was chosen for its accessibility, ease of use, and cost-effectiveness, enabling efficient distribution, however, limitations include potential sampling bias due to reliance on internet access and the variability of self-reported data, particularly for complex questions.

The study utilized a non-validated survey instrument, potentially introducing biases in respondent answers and challenges in translating terms from English to Czech. These limitations indicate the need for future research to employ validated and reliable survey instruments, allowing for comprehensive data collection and facilitating meaningful comparisons within the literature. Finally, study's cross-sectional design limits causal inferences as it captures data at a single point in time, preventing the ability to assess changes or causal relationships over time.

## 5 Conclusion

In summary, this study is the first to highlight the topic of training load monitoring practices among Czech football coaches, demonstrating different approaches to training monitoring and barriers. While most coaches rely on conventional approaches like paper records and training diaries, a significant portion, particularly at elite levels, are beginning to embrace advanced technologies such as specialized software and GPS tracking for data storage and measures of ETL, respectively. However, the difference in access to resources and human resources plays a significant challenge, with many coaches reporting a lack of equipment and personnel as key barriers. Moreover, an incentive for monitoring ITL and physical fitness assessment needs to be encouraged among the coaches. These issues will require efforts from stakeholders across the football community to ensure that coaches at all levels have the support and tools they need to optimize player performance and mitigate injury risks effectively.

Furthermore, the consistent identification of the Football Association (FAČR) as the preferred source of information informs the importance of governing bodies in developing coaching practices and standards. Collaborative initiatives between coaches and the FAČR can facilitate knowledge sharing, promote the adoption of evidence-based methodologies, and ultimately drive the advancement of football coaching in the Czech Republic. By prioritizing education, resource allocation, and collaboration, the football community can work towards a more unified and informed approach to player monitoring, tending to continue growth and success within the sport.

### 5.1 Prospective

In Czech football, coaches generally monitor the training load, however with lack comprehensive information on monitoring ITL and physical tests. Integrating different measures of load monitoring can improve insight into players' performance and reduce fatigue and injury risks. Similarly to findings from Weston (14), our study suggests that in cases where club resources constrain technological use, educating coaches on cost-effective, reliable measures of ITL such as RPE and physical tests can ensure effective load monitoring across all team levels and coaching positions (head coaches, assistant coaches and strength and conditioning). Increasing information about training load monitoring could be achieved through initiatives like workshops, e-books, and other educational resources, especially for the Football Association together with sports scientists to facilitate the educational process.

## Data Availability

The raw data supporting the conclusions of this article will be made available by the authors, without undue reservation.
