# Supplementary material for: Training load and fitness monitoring in Czech football: coach practices and perspectives
Source: Front Sports Act Living. 2025 Jan 31;7:1513573. doi: 10.3389/fspor.2025.1513573 (PMC11825800; doi:10.3389/fspor.2025.1513573)
Supplement: Supplementary file 1 [file Datasheet1.pdf]

Supplementary Material

1 Supplementary Data

S1 - Survey version in Czech (English)

Monitoring zatížení ve fotbale

Rádi bychom vám poděkovali za váš čas při účasti ve společném výzkumném projektu Masarykovy Univerzity a FAČR, který se zaměřuje na monitorování tréninkové zátěže ve fotbale. Tento projekt je součástí našeho úsilí porozumět postojům a praktikám trenérů a odborníků ve světě fotbalu v oblasti load managementu.

Monitoring load in football

We would like to thank you for your time in participating in the joint research project of Masaryk University and FAČR, which focuses on monitoring training load in football. This project is part of our effort to understand the attitudes and practices of coaches and experts in the world of football in the field of load management.

Projekt se zaměřuje na otázky spojené s monitorováním tréninkové zátěže, překážkami, kterým čelíte ve vašem každodenním tréninkovém prostředí, a také na to, jakými způsoby preferujete získávat informace o této problematice.

The project focuses on issues related to training load monitoring, the obstacles you face in your daily training environment, as well as how you prefer to receive information on this issue.

Odhadujeme, že vyplnění tohoto dotazníku vám zabere maximálně 5 minut. Vaše odpovědi jsou pro nás velmi cenné a budou sloužit k lepšímu porozumění výzvám, kterým trenéři a odborníci čelí v oblasti tréninkové zátěže. Pro získání platných a objektivních výsledků potřebujeme odpovědi od všech, tedy i od těch, kteří zatížení žádným způsobem nemonitorují.

We estimate that it will take you a maximum of 5 minutes to complete this questionnaire. Your answers are very valuable to us and will serve to better understand the challenges that coaches and experts face in the field of training load. In order to obtain valid and objective results, we need answers from everyone, including those who do not monitor the load in any way.

Rádi bychom Vás ujistili, že všechny poskytnuté informace budou zpracovány zcela anonymně. Souhlasem s účastí v projektu poskytujete souhlas s využitím dat pro účely této studie, a dále pro účely vzdělávacích aktivit Fotbalové asociace (FAČR), která obdrží data výhradně v souhrnné podobě. V žádném případě nebudou poskytnuté údaje spojeny s konkrétními respondenty. Projekt byl schválen Etickou komisí pro výzkum na Masarykově Univerzitě pod jednacím číslem EKV-2022-054.

We would like to assure you that all information provided will be processed completely anonymously. By agreeing to participate in the project, you consent to the use of data for the purposes of this study, as well as for the purposes of educational activities of the Football Association (FAČR), which will receive the data exclusively in aggregate form. In no case will the data provided be linked to specific respondents. The project was approved by the Ethics Committee for Research at Masaryk University under action number EKV-2022-054.

Pokud máte zájem o zaslání výsledků této studie, máte možnost zadat svůj e-mail na konci dotazníku.

If you are interested in sending the results of this study, you have the option to enter your email at the end of the questionnaire.

Vaše účast v této studii je dobrovolná a v úvodu dotazník vyjadřujete (ne)souhlas s účastí v představeném projektu. Děkujeme vám za vaši spolupráci a cenný příspěvek k našemu výzkumu. Věříme, že společně přispějeme k následnému rozvoji vzdělávání trenérů v ČR.

Your participation in this study is voluntary and at the beginning of the questionnaire you express your (dis)agreement to participate in the presented project. Thank you for your cooperation and valuable contribution to our research. We believe that together we will contribute to the further development of training for coaches in the Czech Republic.

Dotazník vyplňte prosím kompletně, a pouze jednou!

Please complete the questionnaire completely, and only once!

1.Email

2. Souhlas s anonymní účastí ve výše popsaném projektu *

Označte jen jednu elipsu

[ ] Souhlasím

[ ] Nesouhlasím (*Přeskočte na otázku 22*)

Section 1

Základní informace o respondentech (Basic information about respondents)

Tyto informace o vás a o vaší praxi slouží především k třídění a kategorizaci dat.

This information about you and your practice is primarily used to sort and categorize data

3. Věk (age)

4. Pohlaví * (gender)

[ ] Žena

[ ]Muž

[ ] Nechci uvádět

[ ]Jiné

5. Informace o týmu, který aktuálně trénujete *

(Information about the team you are currently coaching)

Zaškrtněte všechny platné možnosti Check all that apply.

	U16	U17	U18	U19	Dospělí (adult)	žádná z uvedených (none of listed)

Muži (Male)						
Ženy (Female)						

6. Trenérské zkušenosti u aktuální kategorie (Coaching experience in the current category)

Zde číslem uveďte, kolik let máte zkušenost s trénováním vaší aktuální kategorie

Enter the number of years of training experience you have in your current category

7. Název soutěže, ve které vámi vedený tým působí

Name of the competition in which your team is participating

8. Jaká je vaše primární pozice v týmu? * (What is your primary position on the team?)

v případě možnosti "Jiná" prosím uveďte (in the case of "Other", please specify)

Označte jen jednu elipsu. Mark only one ellipsis.

[ ]Hlavní trenér/ka Head coach

[ ]Asistent/ka trenéra Assistant coach

[ ]Kondiční trenér/ka Fitness trainer

[ ]Jiné: Other:

9. Jakou trenérskou licencí disponujete? * What coaching license do you have? *

v případě možnosti "Jiná" prosím uveďte in the case of "Other", please specify

[ ]FAČR C

[ ]UEFA C

[ ]UEFA B

[ ]UEFA A

[ ]UEFA Profi

[ ]Jiné: Other:

10. Dosáhl/a jste na titul ve sportovní vědě nebo obdobném oboru souvisejícím se sportem?

např. Bakalář sportu a tělesné výchovy, nebo jiný vzdělávací systém

You have achieved a degree in sports science or a similar related field sports?

e.g. Bachelor of Sports and Physical Education, or another educational system.

[ ]Ano Yes

[ ]Ne No

[ ] Aktuálně studuji I am currently studying

Section 2

Load monitoring ve vaší praxi Load monitoring in your practice

Load managment v rámci fotbalového tréninku zahrnuje systematické sledování a manipulaci s tréninkovým a zápasovým zatěžováním hráče, což představuje například i obyčejný záznam počtu opakování či minutáže ve formě vedení trenérského deníku. Zahrnuje také různé formy testování, sledování parametrů zatížení jako uběhnutá vzdálenost, tepová frekvence, subjektivní vnímání zátěže, hodnocení kvality spánku apod.

Load management within football training includes systematic monitoring and manipulation of the player's training and match load, which represents, for example, a simple record of the number of repetitions or minutes in the form of keeping a trainer's diary. It also includes various forms of testing, monitoring load parameters such as distance traveled, heart rate, subjective perception of load, assessment of sleep quality, etc.

Skrze komplexní monitoring trenéři získávají důležité informace, které podporují optimalizaci výkonnosti a minimalizaci rizika zranění. Tímto procesem se vytváří individuální a evidence-based (=založen na faktech) přístup k rozvoji hráčů.

Through comprehensive monitoring, coaches obtain important information that supports performance optimization and minimizes the risk of injury. This process creates an individual and evidence-based approach to player development.

Následující otázky se budou věnovat tomuto tématu.

The following questions will address this topic.

11. Sledujete ve své praxi tréninkovou/zápasovou zátěž hráčů?

Do you monitor the training/match load of the players in your practice?

(viz výše – zahrnujeme i trenérské deníky, evidenci minutáže, kondiční testování apod.)

(see above - we also include coach's diaries, minutes records, fitness testing, etc.)

[ ] Ano   Yes

[ ] Ne   No

[ ] Ano, ale pouze svým "trenérským okem"   Yes, but only with your "coaching eye "

Load monitoring ve vaší praxi   Load monitoring in your practice

12. Jak často monitorujete zátěž * How often do you monitor load *

v případě možnosti "Jiná" prosím uveďte   in the case of "Other", please specify

[ ] Každý trénink i zápas Every practice and match

[ ]Pouze během utkání   Only during the match

[ ]Pouze během tréninku Only during training

[ ] Jiné: Other:

13. Jak uchováváte informace o zátěži vašich hráčů?

How do you store your players' load information?

v případě možnosti "Jiná" prosím uveďte in the case of "Other", please specify

[ ] Papírový záznam (= tužka + papír) Paper record (= pencil + paper)

[ ]Microsoft Office (Word, Excel...) Microsoft Office (Word, Excel...)

[ ] Specializovaný software (XPS, Goodathlete...) Specialized software (XPS, Goodathlete...)

[ ] Webové rozhraní

[ ] Jiné: Other:

14. Používáte některý z následujících testů pro monitorování stavu sportovce?

Do you use any of the following tests to monitor an athlete's condition?

v případě možnosti "Jiná" prosím uveďte in the case of "Other", please specify

[ ]Testování rychlosti Speed test

[ ]Testování sílys Strenght test

[ ]Testování vytrvalosti Endurance test

[ ]Testování flexibility Flexibility test

[ ]Testování koordinace Coordination test

[ ]Testové baterie pro posouzení celkového stavu sportovce (EUROFIT, FMS, apod.)

Test batteries for assessing the athlete's overall condition (EUROFIT, FMS, etc.)

[ ]Motorické testování FAČR Motor testing FAČR

[ ]Nepoužívám žádné I don't use any test

[ ] Jiné: Other:

15. Používáte některý z následujících prostředků monitorování tréninkové/zápasové zátěže (tréninkový deník, intervaly zatížení/odpočinku, GPS systémy…)?

You use any of the following methods to monitoring training/match loads (training log, load/rest intervals, GPS systems…)?

v případě možnosti "Jiná" prosím uveďte in the case of "Other", please specify

[ ]Trenérský deník (trvání TJ, množství TJ, intervaly zatížení/odpočinku, apod.)

Training diary (duration, sets, load/rest intervals, etc.)

[ ]Data z utkání (minutáž, počet střel, apod.) Match data (minutes, number of shots, etc.)

[ ]GPS data z vest (vzdálenost, rychlosti, akcelerace, decelerace, apod.)

GPS data from the vest (distance, speed, acceleration, deceleration, etc.)

[ ]Nepoužívám žádné I don't use any

[ ] Jiné: Other:

16. Používáte některý z prostředků pro monitorování interních parametrů zatížení?

Are you using any of the internal load monitoring tools?

v případě možnosti "Jiná" prosím uveďte   in the case of "Other", please specify

[ ]Tepová frekvence (typicky sporttestery) Heart rate (typically sports testers)

[ ] Variabilita srdeční frekvence (HRV/mySASY...)

Heart Rate Variability (HRV/mySASY...)

[ ]RPE (subjektivní vnímání záěteže) RPE (subjective perception of exertion)

[ ]TRIMP (tréninkový impuls/kardio zátěž) TRIMP (Training Impulse/Cardio Load)

[ ]ACWL ratio (poměr akutního a chronického zat

ACWL ratio (acute to chronic load ratio)

[ ]Nepoužívám žádné I don't use any

[ ] Jiné: Other:

17. Používáte některý z prostředků pro biochemické monitorování (použití vzorků krve nebo slin)?

Do you use any of the means for biochemical monitoring (using blood or saliva samples)?

v případě možnosti "Jiná" prosím uveďte   in the case of "Other", please specify

[ ]Testování hladiny hormonů. Hormone level testing

[ ]Testování hladiny laktátu   Lactate level testing

[ ]Testování hladiny glukózy   Glucose level testing

[ ] Testování hladiny haemoglobinu Hemoglobin level testing

[ ]Nepoužívám žádné I don't use any

[ ] Jiné: Other:

18. Mimo výše zmíněné, zjišťujete od hráčů (pomocí nějaké škály) některé z uvede

Apart from the above, do you find out from the players (using a scale) any of the listed?

v případě možnosti "Jiná" prosím uveďte   in the case of "Other", please specify

[ ] Nálada Mood

[ ] Kvalita spánku Sleep quality

[ ] Míra únavy Fatigue

[ ] Bolest svalů Muscle pain

[ ] Úroveň stresu Stress level

[ ]Nepoužívám žádné I don't use any

[ ] Jiné: Other:

19. Používáte-li ještě jakýkoliv jiný prostředek/parametr/přístroj/způsob pro sledování zátěže, prosím ve stručnosti uveďte

If you use any other means/parameter/device/method for load monitoring, please state briefly

Section 3

Překážky pro monitorování zátěže ve fotbale

Barriers to exercise monitoring in football

V této sekci nás zajímá Váš názor na to, co v praxi představuje největší překážky pro to, abyste zátěž svých hráčů pravidelně monitorova

In this section, we are interested in your opinion on what in practice represents the biggest obstacles for you to regularly monitor the workload of your players.

20. Jaké z následujících okolností považujete vy osobně za největší překážky pro sledování zátěže ve fotba

v případě možnosti "Jiná" prosím uveďte in the case of "Other", please specify

Which of the following circumstances do you personally consider to be the biggest obstacles to monitoring stress in football?

[ ] Časová náročnost Time constrain

[ ] Nedostatek materiálního vybavení (resp. nedostatek financí na jeho poří

Lack of material equipment (or lack of funds for its acquisition)

[ ] Neorientuji se v problematice I am not familiar with the issue

[ ] Nedostatek personálu pro sběr a vyhodnocení dat

Lack of personnel for data collection and evaluation

[ ] Jiné: Other:

Section 4

Způsob získávání informací o práci se zatížením ve fotbale

A way to get information about working with loads in football

21. Jakým způsobem získáváte informace týkající se monitorování zátěže ve

How do you get information regarding stress monitoring in football?

v případě možnosti "Jiná" prosím uveďte in the case of "Other", please specify

[ ] Youtube

[ ] Sociální sítě. Social networks

[ ] Blogy a webové stránky o fotbale

FAČR/UČFT školení

Blogs and websites about football FAČR/UČFT training

[ ] Tyto informace nevyhledávám I am not looking for this information

[ ] Jiné: Other:

Section 5

To je vše. Děkujeme za Vaši účast Thank you for your participation

22. Je něco, co jsme se vás nezeptali a rádi byste zmínili ohledně sledování zatížení ve fotbale? Používáte věci, které jste nezvládli do předchozích otázek zařadit? Uveďte prosím stručn

Is there anything we haven't asked you that you would like to mention about load monitoring in football? Do you use things that you didn't manage to include in the previous questions? Please be brief.

23. Výsledky chci zaslat na tento mail: I want to send the results to this email:
